# Individual- and area-level socioeconomic inequalities in cancer incidence in the working-age population – a cohort study based on German statutory health insurance data, 2015 to 2019

**DOI:** 10.1186/s12889-025-25890-4

**Published:** 2025-12-09

**Authors:** Simon Brinkwirth, Juliane Tetzlaff, Anja Cengia, Marco Alibone, Benjamin Wachtler, Jens Hoebel, Fabian Tetzlaff

**Affiliations:** 1 Department of Infectious Disease Epidemiology, Postgraduate Training for Applied Epidemiology (PAE), Robert Koch Institute, Berlin, Germany; 2 ECDC Fellowship Programme, Field Epidemiology path (EPIET), European Centre for Disease Prevention and Control (ECDC), Stockholm, Sweden; 3 Department of Medical Sociology, Hannover Medical School, Hannover, Germany; 4 Institute for Applied Health Research Berlin GmbH, Berlin, Germany; 5 Division of Social Determinants of Health, Department of Epidemiology and Health Monitoring, Robert Koch Institute, Nordufer 20, Berlin, 13302 Germany

**Keywords:** Cancer incidence, Socioeconomic inequalities, German index of socioeconomic deprivation, Individual-level socioeconomic inequalities, Area-level socioeconomic inequalities, Working-age population, Germany

## Abstract

**Introduction:**

Cancer is a major public health challenge in Germany with significant socioeconomic inequalities in incidence and mortality. However, there is only limited research on the incidence of diagnosis-specific cancers and related inequalities among socioeconomic groups within the working-age population. This study aims to address this gap by analysing how the incidence of common cancers depends on individual- and area-level socioeconomic characteristics among working-age women and men.

**Methods:**

Using a prospective cohort design based on anonymised German statutory health insurance data, this study examined a cohort of 2.23 million individuals aged 25–67 years over a five-year period (2015–2019). Individual socioeconomic position was assessed using educational attainment and occupational skill levels, while area-level deprivation was determined using a composite socioeconomic index. Incidence rates were estimated per 100,000 person-years at risk, age-standardised to the 2013 European standard population. Hazard ratios were calculated using multilevel Cox proportional hazards models.

**Results:**

The analysis revealed 50,276 newly diagnosed cancer cases during the study period. Lower education, lower occupational skill levels and higher area-level deprivation were associated with a higher incidence of stomach, lung, colorectal, prostate, breast and cervical cancer, but a lower incidence rate of malignant melanoma of the skin. After mutual adjustment of the socioeconomic indicators, higher hazard ratios of lung cancer were found for men with lower educational (HR = 2.8, 95%CI:2.3–3.5) and occupational skill levels (HR = 2.8, 95%CI:2.3–3.5) and for women with lower education (HR = 2.3, 95%CI:1.7–3.1). Lower occupational skill levels in both sexes (women HR = 0.6, 95%CI:0.5–0.7; men HR = 0.7, 95%CI:0.6–0.9) and lower educational levels in men (HR = 0.7, 95%CI:0.6–0.8) were independently associated with a lower risk of malignant melanoma. For area-level deprivation, we observed a higher risk of stomach (women 1.6, 95%CI:1.2-2.0; men HR = 1.3, 95%CI:1.1–1.6) and lung cancer (women HR = 1.3, 95%CI:1.1–1.5; men HR = 1.5, 95%CI:1.3–1.7) in more deprived areas, even after adjusting for individual-level socioeconomic characteristics. In contrast, a higher risk of skin melanoma was observed in less deprived areas after individual-level socioeconomic adjustments (women and men HR = 0.6, 95%CI:0.5–0.7).

**Conclusions:**

Our findings suggest that strategies to prevent cancer in the working-age population should take more account of the unequal structural conditions in which people work and live. The study shows that area-level socioeconomic deprivation has explanatory power for unequal cancer risks beyond the individual characteristics of socioeconomic position.

**Supplementary Information:**

The online version contains supplementary material available at 10.1186/s12889-025-25890-4.

## Introduction

Cancer represents one of the major contributors to morbidity and premature mortality in Germany [1–4]. Many national and international studies have shown that there are considerable socioeconomic inequalities in cancer incidence [1, 5–13], mortality [6, 11, 13–17] and in the length of cancer-free life expectancy [8, 18]. However, previous research so far has scarcely investigated cancer incidence in the working-age population and how it varies depending on individual- and area-level socioeconomic characteristics in this subpopulation.

The study of cancer incidence and associated socioeconomic inequalities in the working-age population is a key topic of high relevance to public health and epidemiological research. This is particularly important given the ageing population, a major challenge for high-income countries in the 21 st century, which has led to a growing imbalance between workers and non-workers and challenging retirement systems. This also holds true for Germany, where the average age of the population in 2021 was five years higher than in 1990 [19]. The working-age population is ageing rapidly, and the mean age of this group is moving closer to the age of onset of most cancers [18, 20]. This group is especially important from a public health perspective as it may still be possible to prevent certain cancers through primary prevention measures. Cancer is a disease that often ends a person’s working life and causes high premature mortality [4, 21].

A recent study based on German health-claims data reports increasing levels of cancer-free life expectancy for the most common cancers in Germany [8]. These developments are due to the decline in cancer incidence and mortality. The overall pattern of inequalities in cancer incidence and mortality by socioeconomic group appears to be comparable for common cancers, but is more pronounced in the working-age population than in older age groups [1, 8, 22]. Socioeconomic inequalities in premature mortality are most pronounced for lung cancer and other preventable cancers, contributed to the stagnation of life expectancy in Germany [3]. Socioeconomic inequalities in behavioural risk factors and work-related exposures have been shown to be two of the main drivers of socioeconomic inequalities in cancer incidence in the majority of high-income countries [6, 12, 20, 23–31]. This highlights the importance of additional studies focusing on socioeconomic inequalities in the incidence of specific preventable cancers in the working-age population to complement previous studies on overall cancer mortality in the general population.

The data available in Germany to analyse socioeconomic inequalities at population level is limited due to the official data-collection practices that do not record cancer incidence or mortality by socioeconomic characteristics [3, 32]. As a result, it is challenging to analyse socioeconomic inequalities in the general or working-age population based on official statistics. Therefore, alternative data sources, such as data from statutory health insurance providers, have to be used in Germany for this purpose [18, 22, 33–36]. Another strategy for overcoming the shortcomings of official statistics is to apply ecological study designs using data at the area level to analyse socioeconomic cancer incidence inequalities across Germany. Up to now, ecological studies have mainly focused on cancer mortality in official statistics [3–5, 17], whereas only few German ecological studies have analysed socioeconomic inequalities in cancer incidence [1, 5]. For this study, we have access to representative cohort data for Germany, which allows us to use indicators of SEP at the individual level in combination with additional regional socioeconomic contextual information. To our knowledge, this is the first multilevel study for Germany which simultaneously analyses individual and area-level socioeconomic inequalities in cancer.

The aim of the study is to expand the existing knowledge on socioeconomic inequality in cancer incidence in the German working-age population. In order to provide additional explanatory power and inform structural prevention strategies, this study draws a comprehensive picture of socioeconomic inequalities that rely on both individual- and area-level inequalities. In addition, we compare the results on individual- and area-level socioeconomic deprivation to investigate the extent to which inequality may be confounded when ecological study designs are used. We focus on common cancers that are sensitive to behavioural risk factors and work-related exposures and are known to be unequally distributed between socioeconomic groups in Germany (e.g. lung and stomach cancer, cancers of the colon and rectum) [1, 5]. Due to the importance of health behaviour and the work and living environment for the pathogenesis of cancer, education and occupational groupings are considered as two of the key characteristics of socioeconomic position at the individual level [20, 26–28]. The following research questions will be investigated:

- How large are socioeconomic inequalities in the incidence of common, preventable cancers at working age based on individual-level socioeconomic position and area-level socioeconomic deprivation?
- To which extent or in which direction do socioeconomic inequalities in cancer incidence differ between the analyses based on individual- and area-level socioeconomic characteristics?
- Is area-level deprivation associated with cancer incidence beyond socioeconomic factors at the individual level?

## Methods

**Fig. 1:**
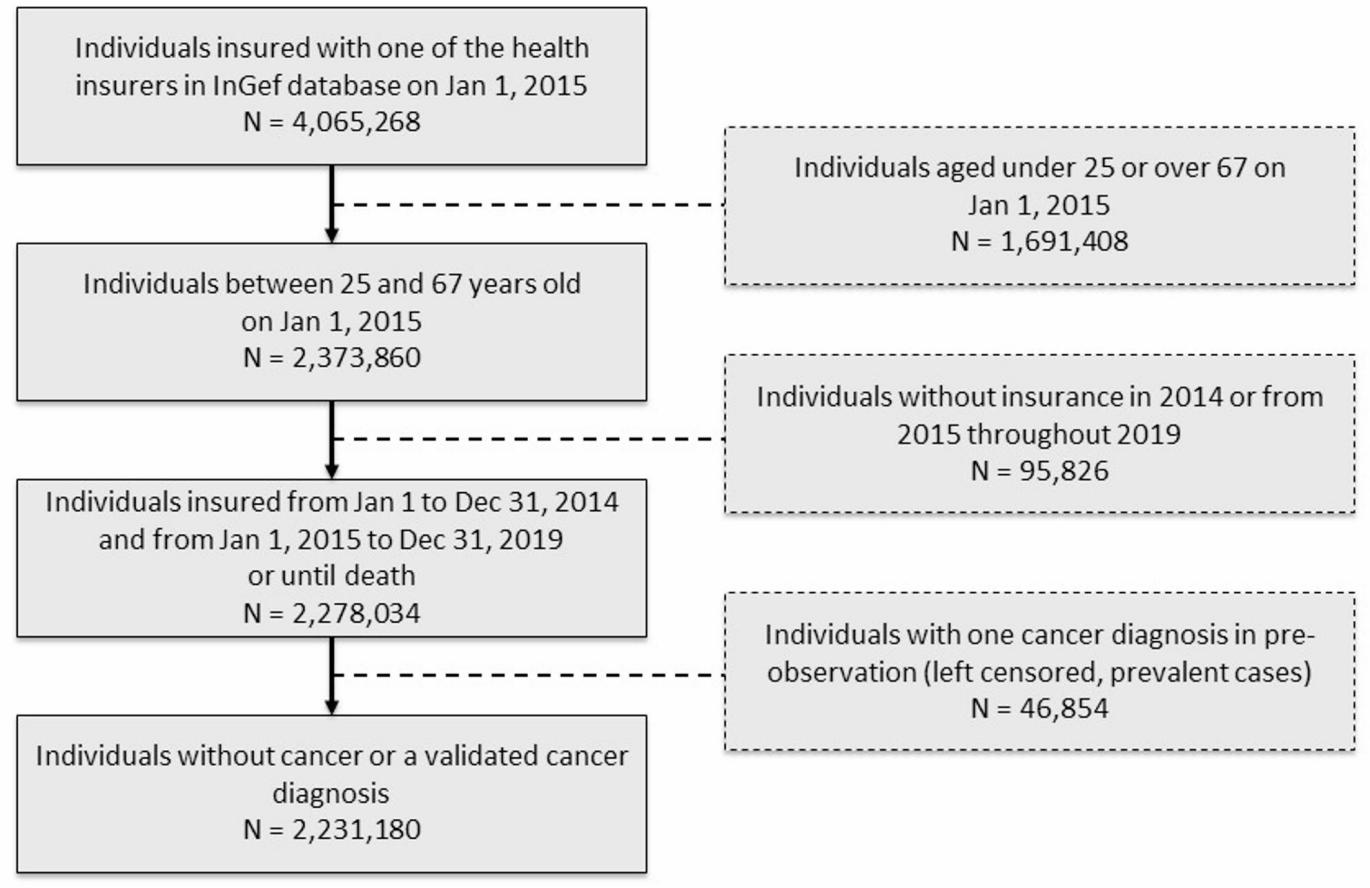
Flow chart of the study population, Germany from 2015 to 2019

This study used a prospective population-based cohort design and was based on anonymised data from the Statutory Health Insurance (SHI) database of the Institute for Applied Health Research Berlin (InGef). Health insurance is compulsory in Germany, and approximately 90% of the population is covered by SHI. The database contains anonymised longitudinal data on approximately nine million individuals covered by company or guild SHI. For our analysis, we used a stratified representative sample of approximately four million insured individuals from the InGef database. This sample was selected to match the age and sex distribution of the German population, ensuring representativeness for the variables of interest [8, 37]. In the German health insurance system, individual insurance is provided through employment, family insurance (i.e., being married to someone who is fully insured), or through the job centre for unemployed individuals. The study population consisted of individuals aged between 25 and 67 on Jan 1, 2015 (Fig. 1). We analysed cancer incidence across an observation period from Jan 1, 2015, to Dec 31, 2019. Data from 2014 were used to exclude spurious incident cases at the start of the observation period by applying a one-year ‘washout’ period. This was done by reviewing insurance records to identify and remove individuals that became incident before the observation period began (old prevalent cases, left censored cases) (Supplement, Figure S1). The dataset included information on sociodemographic characteristics (e.g. age at diagnosis, code of professional activity, place of residence, etc.) and information on cancer diagnoses via International Classification of Diseases, 10th Revision (ICD-10) codes.

### Outcome of interest

The outcome variable was based on the first occurrence of a cancer diagnosis during the observation period. In line with previous German studies on socioeconomic inequalities in cancer incidence [5, 8], the following ICD-10 diagnosis codes were used to define incident cases:

- Stomach cancer (C16).
- Colorectal cancer (C18 – C20).
- Lung cancer (C33 – C34).
- Malignant melanoma of the skin (C43).
- Female breast cancer (C50).
- Cervix uteri (C53).
- Prostate cancer (C61).

The established M2Q validation method [38] with cancer-specific look-back periods [8, 39] was used to identify incident cases in the SHI data. According to this method, diagnoses were either validated by at least two outpatient diagnoses within the four quarters following the quarter of the first diagnosis, or by at least one inpatient diagnosis. The incidence date was set to the date of hospital admission or, in cases of outpatient diagnosis, to the date of the first contact with the diagnosing physician within the validated quarter. In previous analyses, look-back periods of one year have been shown to be feasible for most cancer entities to identify prevalent (left-censored) cases (e.g [8]). Prevalent individuals were excluded from the entity-specific analyses (Fig. 1).

### Individual-level socioeconomic position

Individual-level socioeconomic position (SEP) was assessed using the professional-activity code as described in the Classification of Occupations (KldB 2010) of Germany’s Federal Employment Agency [40]. This code is transmitted via the employer to the health insurance and therefore usually not available for those who are not officially employed over a longer period of time (i.e. those covered under family insurance or unemployed), resulting in missing data in the dataset. Our analysis included two indicators of individual SEP:

1. Formal educational level (education): using the 6th digit (school-leaving qualification) and 7th digit (professional qualification) of the KldB-based professional-activity code, we identified the highest educational level documented in the insurance data. According to the CASMIN (Comparative Analysis of Social Mobility in Industrial Nations) classification [41], the school and professional qualifications were combined and categorised as ‘low’ (CASMIN category 1), ‘middle’ (CASMIN category 2) or ‘high’ (CASMIN category 3) education.
2. Occupational skill level (occupation): based on the 5th digit of KldB-based professional-activity codes [40], the occupational skill level was classified as:

‘unskilled’ - involves simple, routine tasks that require little or no specialised knowledge. These roles generally do not require formal qualifications, or only one year of vocational training, and include unskilled and semi-skilled jobs, as well as one-year regulated training programs;

‘skilled’ - more complex and specialised than unskilled or semi-skilled roles, requiring in-depth specialist knowledge and skills. Typically, these jobs require two to three years of vocational training, equivalent to a vocational qualification from a school or college, or relevant work experience;

‘specialist’ - are significantly more complex, requiring specialised knowledge and skills. These roles often involve planning and control tasks, such as work preparation, resource planning, and quality assurance. The necessary expertise is typically gained through professional development or further education;

or ‘expert’ - very high complexity and demand advanced knowledge and skills. These roles typically include activities such as development, research, diagnostics, knowledge transfer, and leadership within large organisations. To perform these jobs, at least a four-year university education and/or significant work experience is usually required.

To minimise missing values in the individual-level SEP variables, the first KldB that appeared in the data in the period between Jan 1, 2015 and Dec 31, 2019 was used. This value was then adopted for the entire time period. For the education variable, the highest educational qualification documented in this period was used and imputed for the entire observation period.

### Area-level socioeconomic deprivation

We analysed area-level inequalities in cancer incidence, which depict a broader range of (area-level) SEP characteristics than the two indicators of individual occupation and educational level. For this analysis, the insurance data were linked to the German Index of Socioeconomic Deprivation (GISD) [42] using the place of residence at the district level (German ‘Landkreise und kreisfreie Städte’; *n* = 402; territorial status: 2016). In the German administrative system, a district is a mid-level geographical unit situated between municipalities (‘Gemeinden’) and federal states (‘Bundesländer’). Districts typically consist of several municipalities and represent administrative regions with shared local governance. The GISD is based on nine area-based indicators of core dimensions of socioeconomic inequality:

- education: proportions of individuals with a university degree, employees without school qualification, and individuals with incomplete schooling.
- employment: employment rate, gross wage, or salary.
- income: net household income, debt ratio and tax revenue.

The residential districts were categorised into quintiles based on the level of their GISD and were assigned to three deprivation groups (deprivation: low = 1 st quintile; middle = 2nd to 4th quintile; high = 5th quintile). The GISD is published under the CC by 4.0 licence and is available online [43].

### Statistical analysis

Incidence rate was calculated as incidence per 100,000 person-years at risk and was age-standardised to the 2013 EU population (Supplement Table S1) [44]. The time at risk was determined for each person in the dataset and was calculated as the time between cohort entry and cohort exit (until the date of diagnosis, date of death, end of insurance, or end of the study period).

The multivariate analysis is based on a multilevel Cox proportional-hazards model [45] containing the covariates age, area-level deprivation, education and occupation to quantify the effect of SEP on cancer incidence. Districts were used as random intercept. Age was classified into five-year age groups, except for the last age group, which includes 60–67 years. To account for the cluster nature of the data (age groups within districts), the models were adapted as multilevel models with age groups as first-level and districts as second-level units. Individuals with missing data on the independent variables were included in the analysis and categorised as missing information. Individuals who could not be followed across the full observation period, e.g. due to a change of insurance, were right-censored to the last available date. We followed a two-step analysis strategy: First, in the sex-specific Models 1a to 1f we estimated the effect on the SEP indicators on cancer incidence adjusted for age. Model 1a includes education and adjusts for age among men, whereas Model 1f includes area-level socioeconomic deprivation and adjusts for age among women. In the second step, the three SEP indicators, individual-level education, occupational skill level and area-level deprivation, were analysed together in a respective sex-specific model. All results were reported with 95% confidence intervals. R (Version 4.2.1) was used to analyse the data and visualise the results. To assess multicollinearity among covariates in regression models, the vif() function from the car package in R was used [46]. For models including categorical predictors with multiple degrees of freedom, we relied on the scaled version of the Generalised Variance Inflation Factor (GVIF), as recommended by Fox and Monette [47]. This allows for consistent comparison across predictors regardless of dimensionality. A VIF value of more than five indicates problematic multicollinearity. According to the authors, values below this threshold do not indicate relevant multicollinearity [47].

### Data anonymisation and ethical considerations

In accordance with Sect. 284 in conjunction with Sects. 70 and 71 of the German Social Code, Book V (SGB V), reimbursements for healthcare services are transmitted directly from the service providers to specialised data centres of the health insurance providers. All data are anonymised before being entered into the InGef database and are therefore no longer considered personal data in accordance with Sect. 67 § 2 SGB X. In conjunction with Article 4 no. 1 of the General Data Protection Regulation (GDPR), their use for scientific research purposes complies with German law. Additional approval by an ethics committee was not required.

## Results

The study included 2,231,180 individuals with a total of 10,594,114 person-years at risk during the observation period from 2015 to 2019. During this period, a total of 50,276 cancer cases were diagnosed with one of the defined cancers. The characteristics of the study population, including individual educational level (education), occupational-skill level (occupation) and area-level socioeconomic deprivation (deprivation) are displayed in Table 1.

**Table 1:** Characteristics of study population, Germany from 2015 to 2019

	Total		Women		Men	
	(*N*)	(%)	(*N*)	(%)	(*N*)	(%)
**Population**	2,231,180		1,107,022		1,124,158	
Person-years at risk	10,594,114		5,259,638		5,334,476	
Number of first level units^a^	36,180		18,090		18,090	
Number of second level units^b^	402		402		402	
**Cancer cases**	50,276	100	28,072	100	22,204	100
Stomach cancer	2,058	4	718	3	1,340	6
Colorectal cancer	8,653	17	3,922	14	4,731	21
Lung cancer	7,303	15	3,039	11	4,264	19
Malignant melanoma of the skin	7,390	15	4,089	15	3,301	15
Female breast cancer	14,208	28	14,208	50	-	
Cervix uteri	2,096	4	2,096	7	-	
Prostate cancer	8,568	17	-		8,568	39
Education*						
Low	590,878	26	211,695	19	379,183	34
Middle	735,339	33	386,562	35	348,777	31
High	217,590	10	96,391	9	121,199	11
Missing information	687,373	31	412,374	37	274,999	24
Occupation*						
Unskilled	223,384	10	117,669	11	105,715	9
Skilled	1,043,295	47	493,010	45	550,285	49
Specialist	237,745	11	88,746	8	148,999	13
Expert	185,626	8	69,168	6	116,458	10
Missing Information	541,130	24	338,429	31	202,701	18
Area-level socioeconomic deprivation*						
High	332,759	15	161,791	15	170,968	15
Middle	1,292,828	58	642,351	58	650,477	58
Low	560,921	25	280,973	25	279,948	25
Missing Information	44,672	2	21,907	2	22,765	2

^a^Product of the number of age groups (*n* = 9), districts (*n* = 402), observation years (*n* = 5), and sexes (*n* = 2)

^b^Number of German districts or ‘Landkreise und kreisfreie Städte‘; n = 402; territorial status 2016

*The %-column is presented as a percentage of the total population and further disaggregated by sex

### Cancer incidence by education

**Fig. 2:**
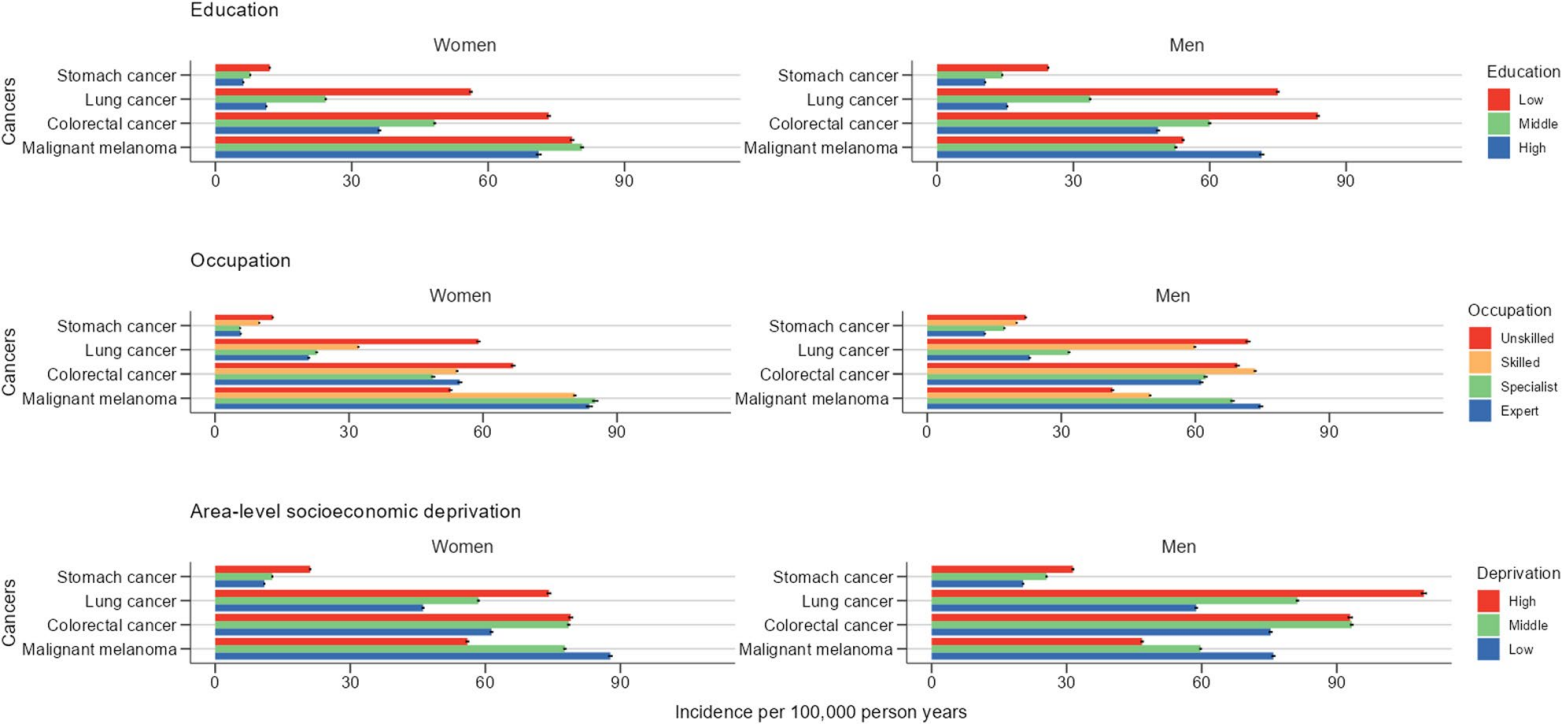
Cancer incidence rates by education, occupation and deprivation in the German statutorily health-insured working age-population, Germany 2015–2019

Higher levels of education were consistently associated with a significantly lower incidence of almost all cancers, including stomach, lung, colorectal, prostate, breast and cervical cancers. A reverse association was observed for malignant melanoma of the skin, where a higher level of education was associated with a significantly higher incidence in both men and women (Fig. 2, Supplement Figure S2).

### Cancer incidence by occupation

Similar patterns were observed when considering the occupational skill level. Lower occupational skill levels were linked to a significantly higher incidence of cancers such as stomach, lung, colorectal and cervix uteri. An exception to this general pattern was again found in skin melanoma, for which incidence rates rose with increasing occupational skill level. Prostate cancer incidence varied only slightly by occupational skill level, with the lowest incidence among ‘unskilled’ workers. Female breast cancer incidence appeared largely unaffected by occupational skill level (Fig. 2, Supplement Figure S2).

### Cancer incidence by area-level socioeconomic deprivation

Inequalities in cancer incidence were apparent when studied by area-level socioeconomic deprivation. Higher levels of socioeconomic deprivation were associated with a higher incidence of stomach, lung, colorectal, prostate, breast and cervical cancer. As with the aforementioned association between individual educational and occupational skill level, we observed a reverse gradient, whereby higher incidence rates of malignant melanoma of the skin were associated with lower deprivation.

Gradients based on area-level deprivation were steeper than those based on individual educational and occupational level for malignant melanoma and stomach cancer in women (Fig. 2, Supplement Figure S2).

### Incidence risk by educational level

Table 2 shows the results of the Cox proportional hazards ratio (HR) models to estimate incidence risks by socioeconomic factors both with adjustment for age only (Model 1a to 1f) and with additional adjustment for all above-mentioned socioeconomic indicators (Model 2a and 2b). Women with middle and low levels of education had significantly higher risks of lung cancer compared to women with high levels of education. These associations remained significant even after adjusting for occupational skill level and area deprivation (middle education HR: 1.6, 95% CI: 1.2–2.2; low education HR: 2.3, 95% CI: 1.7–3.1). Similar inequalities were found for men. For colorectal cancer, a significant higher incidence risk was only observed for women with a low education level compared to those with a high education level in the fully adjusted model (HR: 1.3, 95% CI: 1.1–1.6). In contrast, we found a 40% lower incidence risk for malignant melanoma of the skin among men with a low education level and a 28% lower risk among men with a middle educational level compared to men with a high educational level. Further controlling for occupation and area-level socioeconomic deprivation weakened this association, but a clear, reversed gradient persisted (middle education HR: 0.8, 95% CI: 0.7–0.9; low education HR: 0.7, 95% CI: 0.6–0.8). No association between incidence risk for malignant melanoma and education was found in women (Table 2).

**Table 2:** Hazard ratios (95% CI) of cancer by socioeconomic factors among the statutorily health-insured working age-population, Germany 2015–2019

	Stomach cancer		Colorectal cancer		Lung cancer		Malignant melanoma of the skin	
	Step 1 HR and 95% CI	Step 2 HR and 95% CI	Step 1 HR and 95% CI	Step 2 HR and 95% CI	Step 1 HR and 95% CI	Step 2 HR and 95% CI	Step 1 HR and 95% CI	Step 2 HR and 95% CI
**Women**	*n* = 718	*n* = 718	*n* = 3,**922**	*n* = 3,**922**	*n* = 3,**039**	*n* = 3,**039**	*n* = 4,**089**	*n* = 4,**089**
**Education**	**Model 1a**	**Model 2a**	**Model 1a**	**Model 2a**	**Model 1a**	**Model 2a**	**Model 1a**	**Model 2a**
Low	1.3 (0.8–1.9)	1.0 (0.6–1.6)	1.2 (1.0–1.4.0.4)	1.3* (1.1–1.6)	2.4* (1.8–3.2)	2.3* (1.7–3.1)	0.9 (0.8–1.0.8.0)	1.1 (0.9–1.2)
Medium	1.0 (0.7–1.6)	0.8 (0.5–1.3)	1.0 (0.9–1.2)	1.2 (1.0–1.4.0.4)	1.6* (1.2–2.2)	1.6* (1.2–2.2)	1.0 (0.9–1.2)	1.1 (1.0–1.3.0.3)
High (ref)								
Missings	1.6* (1.1–2.3)	1.5 (0.9–2.4)	1.2* (1.0–1.4.0.4)	1.4* (1.1–1.7)	2.7* (2.0–3.5.0.5)	2.3* (1.7–3.3)	0.7* (0.7–0.8)	0.9 (0.8–1.1)
**Occupation**	**Model 1b**		**Model 1b**		**Model 1b**		**Model 1b**	
Unskilled	1.7* (1.0–2.8.0.8)	1.6 (0.9–2.8)	0.9 (0.7–1.0.7.0)	0.7* (0.6–0.9)	1.9* (1.4–2.4)	1.3 (1.0–1.7.0.7)	0.6* (0.5–0.7)	0.6* (0.5–0.7)
Skilled	1.5 (1.0–2.4.0.4)	1.6 (0.9–2.6)	0.8* (0.7–1.0.7.0)	0.8* (0.6–0.9)	1.3 (1.0–1.6.0.6)	1.0 (0.7–1.3)	0.9 (0.8–1.0.8.0)	0.9 (0.8–1.0.8.0)
Specialist	0.9 (0.5–1.7)	1.0 (0.5–1.8)	0.8 (0.7–1.0.7.0)	0.8* (0.6–1.0.6.0)	1.0 (0.8–1.4)	0.9 (0.6–1.2)	1.0 (0.8–1.2)	1.0 (0.8–1.1)
Expert (ref)								
Missings	1.8* (1.1–2.9)	1.2 (0.7–2.1)	0.9 (0.8–1.1)	0.8* (0.6–0.9)	1.8* (1.4–2.3)	1.1 (0.8–1.5)	0.7* (0.6–0.8)	0.8* (0.6–0.9)
**Area-level deprivation**	**Model 1c**		**Model 1c**		**Model 1c**		**Model 1c**	
High	1.6* (1.2–2.1)	1.6* (1.2–2.0.2.0)	1.1 (0.9–1.3)	1.1 (0.9–1.3)	1.3* (1.1–1.5)	1.3* (1.1–1.5)	0.6* (0.5–0.7)	0.6* (0.5–0.7)
Medium	1.1 (0.9–1.4)	1.1 (0.9–1.3)	1.1 (1.0–1.3.0.3)	1.1 (1.0–1.3.0.3)	1.1 (1.0–1.3.0.3)	1.1 (1.0–1.3.0.3)	0.8* (0.7–0.9)	0.8* (0.8–0.9)
Low (ref)								
Missings	1.8 (0.7–4.3)	1.7 (0.7–4.2)	1.5 (0.6–4.2)	1.5 (0.6–4.2)	1.2 (0.7–2.3)	1.2 (0.7–2.2)	0.9 (0.5–1.7)	1.0 (0.5–1.7)
**Men**	*n* = 1,**340**	*n* = 1,**340**	*n* = 4,**731**	*n* = 4,**731**	*n* = 4,**264**	*n* = 4,**264**	*n* = 3,**301**	*n* = 3,**301**
**Education**	**Model 1d**	**Model 2b**	**Model 1d**	**Model 2b**	**Model 1d**	**Model 2b**	**Model 1d**	**Model 2b**
Low	1.5* (1.1–2.0.1.0)	1.3 (0.9–1.7)	1.1 (1.0–1.3.0.3)	1.1 (0.9–1.3)	2.8* (2.3–3.5)	2.0* (1.6–2.5)	0.6* (0.5–0.7)	0.7* (0.6–0.8)
Medium	1.3 (1.0–1.7.0.7)	1.1 (0.8–1.5)	1.1 (0.9–1.2)	1.1 (0.9–1.2)	1.9* (1.5–2.4)	1.4* (1.1–1.8)	0.7* (0.6–0.8)	0.8* (0.7–1.0.7.0)
High (ref)								
Missings	1.8* (1.4–2.4)	1.5* (1.1–2.2)	1.2* (1.1–1.4)	1.1 (0.9–1.3)	3.8* (3.0–4.7.0.7)	2.4* (1.8–3.1)	0.7* (0.6–0.7)	0.6* (0.5–0.8)
**Occupation**	**Model 1e**		**Model 1e**		**Model 1e**		**Model 1e**	
Unskilled	1.6* (1.2–2.2)	1.4 (1.0–1.9.0.9)	1.1 (0.9–1.3)	1.0 (0.9–1.2)	2.8* (2.3–3.5)	2.0* (1.6–2.5)	0.6* (0.5–0.7)	0.7* (0.6–0.9)
Skilled	1.4* (1.1–1.8)	1.3 (1.0–1.7.0.7)	1.1 (1.0–1.2.0.2)	1.0 (0.9–1.2)	2.3* (1.9–2.7)	1.7* (1.4–2.1)	0.7* (0.6–0.7)	0.8* (0.7–0.9)
Specialist	1.3 (1.0–1.7.0.7)	1.2 (0.9–1.6)	1.0 (0.9–1.1)	1.0 (0.8–1.1)	1.3* (1.1–1.7)	1.1 (0.9–1.4)	0.9 (0.8–1.0.8.0)	1.0 (0.9–1.2)
Expert (ref)								
Missings	1.8* (1.4–2.3)	1.3 (0.9–1.8)	1.2* (1.1–1.4)	1.1 (0.9–1.3)	3.2* (2.7–3.9)	1.9* (1.5–2.4)	0.8* (0.7–0.9)	1.1 (0.9–1.3)
**Area-level deprivation**	**Model 1f**		**Model 1f**		**Model 1f**		**Model 1f**	
High	1.4* (1.1–1.6)	1.3* (1.1–1.6)	1.0 (0.9–1.2)	1.0 (0.9–1.2)	1.5* (1.3–1.7)	1.5* (1.3–1.7)	0.6* (0.5–0.6)	0.6* (0.5–0.7)
Medium	1.2* (1.0–1.3.0.3)	1.2 (1.0–1.9.0.9)	1.1 (1.0–1.3.0.3)	1.1 (1.0–1.3.0.3)	1.3* (1.1–1.4)	1.2* (1.1–1.4)	0.7* (0.7–0.8)	0.8* (0.7–0.8)
Low (ref)								
Missings	1.3 (0.8–2.2)	1.2 (0.7–2.1)	1.4 (0.7–2.9)	1.4 (0.6–2.9)	1.7* (1.0–2.9.0.9)	1.6 (1.0–2.6.0.6)	0.7 (0.4–1.2)	0.8 (0.4–1.3)

Step 1: sex-specific models 1a to 1f are six independently estimated models in which each of the three SEP indicators used was separately adjusted for age

Step 2: sex-specific models 2a and 2b with adjustment for age and mutual adjustment for education, occupation and area-level socioeconomic deprivation

*statistically significant (p-value ≤ 0,05)

Table S2 shows the results of the hazard ratios for sex-specific cancers by socioeconomic factors (Supplement Table S2). Women with low levels of education had a significantly lower HR for female breast cancer compared to women with higher levels of education in the adjusted model (HR: 0.9, 95% CI: 0.8–1.0.8.0). For cervical cancer, women with low and middle levels of education had significantly higher HRs compared to women with high levels of education in the adjusted models (low education HR: 1.1, 95% CI: 1.2–1.8; middle education HR: 1.4, 95% CI: 1.2–1.8). Men with low and middle levels of education had significantly lower incidence risk for prostate cancer compared to men with high levels of education in the adjusted models (low education HR: 0.8, 95% CI: 0.7–0.9; middle education HR: 0.9, 95% CI: 0.8–1.0.8.0).

### Incidence risk by occupational level

For colorectal cancer, women in unskilled, skilled and specialist occupations exhibited significantly lower HRs compared to those in expert occupations. This pattern remained stable in the adjusted model (unskilled HR: 0.7, 95% CI: 0.6–0.9; skilled HR: 0.8, 95% CI: 0.6–0.9; specialist HR: 0.8, 95% CI: 0.6–1.0). For malignant melanoma of the skin, a considerably lower incidence risk was observed in men and women in unskilled occupations and in men in skilled occupations compared to the expert group. This association was also evident after the adjustment for education and area deprivation. In contrast, men in unskilled and skilled occupations exhibited significantly higher HRs for lung cancer compared to men in expert occupations. This association also persisted after adjustment (skilled HR: 1.7, 95% CI: 1.4–2.1; unskilled HR: 2.0, 95% CI: 1.6–2.5) (Table 2).

Regarding sex-specific cancers (Supplement Table S2), women in unskilled occupations had significantly lower hazard ratios for breast cancer (HR: 0.9, 95% CI: 0.8–1.0) compared to those in expert occupations in the adjusted model. For cervical cancer, women in unskilled occupations had significantly higher HRs compared to women in expert occupations in the adjusted model (HR: 1.3, 95% CI: 1.0–1.7.0.7).

### Incidence risk by area-level socioeconomic deprivation

Higher levels of area-level socioeconomic deprivation were significantly associated with a higher incidence risk for stomach and lung cancer in both sexes. After adjusting for the individual-level indicators of SEP, this association was weaker but still significant in Model 2 for women (stomach cancer HR: 1.6, 95% CI: 1.2–2.0; lung cancer HR: 1.3, 95% CI: 1.1–1.5) and men (stomach cancer HR: 1.3, 95% CI: 1.1–1.6; lung cancer HR: 1.5, 95% CI: 1.3–1.7). Again, for malignant melanoma of the skin a reverse gradient was found with significantly lower HRs in areas with high deprivation, with comparable levels of incidence risk in Model 1 and Model 2 for both sexes (high deprivation HR: 0.6, 95% CI: 0.5–0.7; middle deprivation HR: 0.8, 95% CI: 0.7–0.9) (Table 2).

Concerning sex-specific cancers (Supplement Table S2), women residing in highly deprived areas exhibited significantly higher hazard ratios for cervical cancer compared to those living in areas of middle or low deprivation in the adjusted model. The HR for middle deprivation was 1.2 (95% CI: 1.0–1.4), and for low deprivation, it was 1.4 (95% CI: 1.1–1.6). For breast and prostate cancer, no associations between area-level socioeconomic deprivation and incidence risk were found, either in the models controlling for area-level socioeconomic deprivation only or in the models mutually adjusting for individual and regional determinants of SEP.

### Variance inflation factor

We performed the Variance Inflation Factor to test against multicollinearity in our models. The values for the all regression models ranged between 1.00 and 1.42 (Supplement Table S3). The values we measured in our models are considerably below the threshold value of 5, which indicates little to no multicollinearity between the used covariates.

## Discussion

To our knowledge, this is the first multilevel study for Germany analysing both individual-based and area-level socioeconomic inequalities in diagnosis-specific cancer incidence focusing especially on the working-age population. The study shows substantial inequalities in cancer incidence in the working-age population, mostly to the disadvantage of people with low SEP or living in regions with high socioeconomic deprivation. This holds for almost all the cancers considered, which indicates that the working-age population should be a greater focus in public-health efforts that aim to reduce socioeconomic inequalities in cancer incidence. These inequalities are evident for both individual-level indicators of SEP and area-level socioeconomic deprivation. Area deprivation shows associations with cancer incidence beyond individual-level SEP, which was found for lung and stomach cancer and malignant melanoma of the skin among women and men. However, the socioeconomic patterns vary among these cancers. Malignant melanoma shows a reverse social gradient, i.e. has a higher incidence in higher socioeconomic groups and less deprived areas. Moreover, the correlation of individual- and area-level indicators of SEP with cancer incidence varies in magnitude and direction for the different cancers: for individual-level determinants, lower levels of education and occupation were associated with a higher risk of being diagnosed with lung cancer. This is also true for colorectal cancer among women with lower occupational levels. In contrast to the patterns observed above, lower levels of education and occupation were associated with a lower risk of malignant melanoma. For area-level socioeconomic deprivation, we observed a higher risk of stomach and lung cancer among residents of more deprived districts. Conversely, a lower incidence risk of malignant melanoma of the skin was observed in more deprived districts.

Cancer is well known to have a huge impact on the life of affected individuals [4, 21], including their working lives. This is caused by the two-directional relationship between cancer and SEP: on the one hand, low SEP causes poorer health opportunities and can lead to increased risks of cancer [48]. On the other hand, cancer usually represents a life-changing diagnosis that interrupts (or even ends) the working life, and can lead to socioeconomic downward mobility [49]. The strong socioeconomic inequalities in cancer found in this study suggest that individuals of working age with lower SEP might be disadvantaged in more ways than one: in terms of their health and in terms of their chances to maintain their SEP. This is especially relevant if, as in Germany, the sickness benefit and disability pension depend on previous income. Future job opportunities can also be significantly affected by successful rehabilitation. This all may lead to a double burden and make them even more vulnerable than the group of older retired people, who may be less likely to experience social downward mobility as a consequence of cancer. This shows that the working-age population should be a greater focus in future studies analysing socioeconomic inequalities in cancer incidence at population level.

Our results are in line with previous national and international studies focusing on socioeconomic inequalities in cancer incidence or cancer mortality among the general population based on area-level socioeconomic deprivation [1, 3–6, 9–11] and on individual-level SEP [6, 13, 16, 18]. These studies also found clear inequalities, which usually show the ‘typical’ gradient of higher incidence or mortality rates in regions or people with lower levels of SEP. In our study, consistently higher incidence risks were found for cancers of the lung, stomach and colon in individuals with lower SEP and individuals living in highly deprived districts than in less deprived districts [1, 8]. For these cancers, lifestyle-related risk factors such as tobacco and alcohol consumption or obesity are often discussed as the most important factors for an increased incidence risk [5, 8, 20]. This is particularly true for people with low SEP, who often exhibit considerably higher rates of tobacco use and obesity [12, 23–26, 28–30, 50], from which their higher incidence risk and the socioeconomic inequalities found in our study can be derived. For Germany, there are few studies that analyse socioeconomic inequalities in malignant melanoma. Comparable results to those in our study have been reported on the basis of health insurance data [8] and aggregated official data [5]. A systematic review of international studies suggests that the reverse association between SEP or socioeconomic deprivation and the incidence of malignant melanoma could be partly explained by lifestyle-related risk factors such as recreational sun exposure and tanning.

 [31]. While this inverse association is characteristic for malignant melanoma, our analysis regarding cervical cancer revealed a higher incidence among women with lower SEP [8] and higher socioeconomic deprivation [5, 51]. Similarly, the review by Mihor and colleagues supports this finding [6], demonstrating that both regional deprivation indices and individual SEP characteristics have independent but comparable effect sizes for the risk of cervical cancer. It can therefore be assumed that a substantial proportion of the unexplained variance could also be attributed to regional socioeconomic deprivation, which is consistent with our findings. For breast cancer, an reverse gradient has been reported internationally [6]. There is also some evidence of this in Germany [5, 8]. However, our study did not find clear evidence for this reverse gradient. We found a slightly higher occurrence of breast cancer with increasing levels of education. When controlling for all socioeconomic characteristics, no inequalities were found among women with high and low SEP. These differences may also be due to the use of a different age cut-off, as we focus on the working age population, while previous studies consider the entire age range. However, our study is the first to shed light on the separate effects of individual characteristics of SEP and regional socioeconomic deprivation, and reveals independent contributions of all analysed individual- and area-level socioeconomic determinants.

International studies have shown that cancer screening programmes have the potential to reduce cancer mortality in the long run. However, they may also exacerbate health inequalities, as individuals with a lower SEP are less likely to be reached by these programmes than those with a higher SEP [52–56]. This could have led to an underestimation of inequalities in our study. However, based on previous studies, it must be assumed that cancer screening has some potential to reduce socioeconomic inequalities in mortality, particularly because cancer mortality is the largest contributor to overall socioeconomic inequalities in mortality. This potential, however, critically depends on ensuring that individuals with lower SEP are effectively reached by screening programmes, which has not consistently been achieved to date. This is particularly relevant for individuals between the ages of 40 and 74 [3]. In contrast, primary cancer prevention through upstream approaches—such as national tobacco control measures (advertising bans, taxation, etc.)—offers a broader potential to significantly reduce health inequalities by addressing major risk factors for cancer incidence and mortality at the societal level.

For Germany, the data situation remains a limiting factor for analysing socioeconomic inequalities in cancer incidence in more detail. Depending on the data source, either information on SEP is lacking in the cancer registry data [20], or individuals with higher SEP are underrepresented in statutory health insurance data [39, 57]. In order to bridge this data gap, previous studies focusing on socioeconomic inequalities in cancer used either ecological study designs without individual-level SEP data [1, 5] or individual-level health insurance data that, however, contained information on the individual-level SEP only [8, 18, 22, 58]. Both approaches have their advantages and limitations. On the one hand, when using ecological study designs, it is only possible to analyse the association between cancer risk and area-level socioeconomic indicators but not on causality. On the other hand, the underrepresentation of individuals with high SEP in statutory health insurance data could lead to an underestimation of socioeconomic inequalities in cancer incidence at the individual level. Our multilevel study combines both approaches to estimate independent effects of individual- and area-level socioeconomic determinants on the risk of cancer in Germany.

The results show that SEP characteristics of the individual and area-level information on socioeconomic deprivation have an independent significant impact on the cancer incidence risk. Since the information on the individual educational and occupational level directly describes a person’s SEP, we expected the effect of this individual characteristics on cancer risk to be greater than that of socioeconomic deprivation at the area level. This was not always the case, as area-level socioeconomic deprivation has a considerable impact on incidence for most of the cancers considered. The GISD reflects not only an average SEP of a region’s population, but also aspects of a region’s economy and socioeconomic context, such as educational and employment opportunities, which can shape the living conditions in the region. The additional effect of area-level socioeconomic inequalities is also demonstrated by the fact that their effect is still evident after controlling for the individual characteristics of education and occupation in different cancers. This suggests that future studies should also use SEP characteristics at regional level in addition to the individual SEP characteristics, as they can provide additional explanatory power for the incidence of cancer.

### Strengths and limitations

We used a sample of data from a statutory health insurance database, which was representative of German general population in terms of age and sex. The main advantage of using this kind of data for studies investigating health inequalities is that the individual insurance histories offer precise information on health outcomes, as well as data on the SEP, including education and occupation extracted from the KldB-based professional-activity code. A particular strength of this study is the additional linkage to area-level socioeconomic deprivation data, which could not be done based on the data of the official cancer registries in previous studies. Our findings therefore provide a deeper understanding of socioeconomic inequalities in the incidence of cancer within the working population.

A limitation is that the occupation code reported by the employer to the social insurance system is rarely updated. This applies in particular to people who have been employed by the same employer for a longer period. It is therefore possible that the individual-level inequalities for older workers may be underestimated, as professional qualifications were not fully reported by the employer. Furthermore, we have no information on individual-level SEP for individuals who were unemployed during the observation period. In particular, individuals with a lower SEP may have been more likely to be excluded for this reason, because they are disproportionately affected by unemployment. We used the imputation of information on educational attainment and occupational skill levels provided by the KldB for the entire observation period to minimise the problems that could arise from missing values and the sole use of SEP information at cohort entry. Even though we applied this approach, the percentage of missing information on education and occupation remains quite high. In addition, privately insured individuals are not included in the data; this selection bias could also have influenced our results. Furthermore, the potential underrepresentation of both lower and higher SEP individuals may have affected our results. Both would lead to an underestimation of cancer inequality, as the variability of the inequality measure would decrease and the estimates would be shifted towards the mean. Nevertheless, there are enough cases in the marginal categories in our database to provide sufficient variance for the estimation of inequalities between SEP groups. This might have resulted in a more conservative estimates of health disparities within our study. Lastly, in the multilevel Cox models we only considered age, sex, education, occupation, and area-level socioeconomic deprivation. Other factors, such as health-seeking behaviours and unhealthy lifestyles, which likely mediate the relationship between SEP and cancer risk, were not captured in the insurance data and should be considered when interpreting the results. In addition, the simultaneous use of correlated variables such as education and occupation could lead to multicollinearity. Therefore, in our analyses (Model 1a to Model 1f), we first examined the single effect of each socioeconomic variable on cancer incidence. Only in Model 2 all socioeconomic variables were included simultaneously in one regression. From this model, it can be concluded that the socioeconomic variables were only slightly to moderately confounded with each other, as the estimates in the models changed only slightly from Model 1 to Model 2. In addition, the values for the variance inflation factor in all regression models are considerably below the threshold value for the VIF, which is a good indicator of little to no multicollinearity. However, the same was shown by a methodological analysis by Geyer et al. who recommend avoiding the exchange of different SEP indicators and utilising the mutual control of all SEP dimensions. This study also found a low to moderate correlation between income, education and occupational status for the German population [59]. Considering the points mentioned above, it seems reasonable to analyse different aspects of SEP, each with its own explanatory power. Finally, we conducted a sensitivity analysis using a complete case approach to examine the extent to which missing data in our SEP variables affected the results. The analysis without missings revealed no noticeable changes in the regression estimators, indicating that our findings can be considered robust (Supplement Tables S4 and S5).

## Conclusion

Against the backdrop of an ageing working population and the increasing imbalance between the number of people in and out of work, maintaining the health of the working-age population into old age and reducing health inequalities in the labour force is becoming a key public-health issue. The strong socioeconomic inequalities in the incidence of common cancers found in this study suggest that the working-age population should be a greater focus in future public-health efforts, such as more targeted cancer-prevention strategies. In this respect, our findings indicate that strategies to prevent cancer in working-age people should focus more on the unequal structural conditions in which they work and live.

## Supplementary Information

Supplementary Material 1.

## Data Availability

No datasets were generated or analysed during the current study.
