# Supplementary material for: Individual- and area-level socioeconomic inequalities in cancer incidence in the working-age population – a cohort study based on German statutory health insurance data, 2015 to 2019
Source: BMC Public Health. 2025 Dec 9;26:119. doi: 10.1186/s12889-025-25890-4 (PMC12781635; doi:10.1186/s12889-025-25890-4)
Supplement: Supplementary file 1 — Supplementary Material 1. [file 12889_2025_25890_MOESM1_ESM.docx]

Supplement: Individual- and areal-level socioeconomic inequalities in cancer incidence in the working-age population – A cohort study based on German statutory health insurance data, 2015 to 2019

Simon Brinkwirth^1,2^, Juliane Tetzlaff^3^, Anja Cengia^4^, Marco Alibone^4^, Benjamin Wachtler^5^, Jens Hoebel^5+^, Fabian Tetzlaff^5+*^

^1^ Department of Infectious Disease Epidemiology, Postgraduate Training for Applied Epidemiology (PAE), Robert Koch Institute, Berlin, Germany

^2^ ECDC Fellowship Programme, Field Epidemiology path (EPIET), European Centre for Disease Prevention and Control (ECDC), Stockholm, Sweden

^3^ Medical Sociology Unit, Hannover Medical School, Hannover, Germany

^4^ Institute for Applied Health Research Berlin GmbH, Berlin, Germany

^5^ Division of Social Determinants of Health, Department of Epidemiology and Health Monitoring, Robert Koch Institute, Berlin, Germany

+ Shared senior authorship

*Corresponding author

Dr. Fabian Tetzlaff

Robert Koch Institute

Division of Social Determinants of Health

Nordufer 20

13302 Berlin

Germany

[TetzlaffF@rki.de](mailto:TetzlaffF@rki.de)

Table of Contents

Figure S1: Timeline of cohort entry and observation period

Figure S2: Sex-specific cancer incidence by education, occupation and deprivation in the German statutorily health-insured working age-population, Germany 2015-2019

Table S1: Distribution of the 2013 European Standard Population (ESP)

Table S2: Hazard ratios (95% CI) of sex-specific cancer by socioeconomic factors among the working-age population with statutory health insurance, Germany 2015-2019

Table S3: Variance Inflation Factor of all regression models

Table S4: Hazard ratios (95% CI) of cancer by socioeconomic factors among the statutorily health-insured working age-population, Germany 2015-2019 (Complete Cases Analysis)

Table S5: Hazard ratios (95% CI) of sex-specific cancer by socioeconomic factors among statutorily health-insured working age-population, Germany 2015-2019 (Complete Cases Analysis)

Table S6: Variance Inflation Factor of all regression models (Complete Cases Analysis)


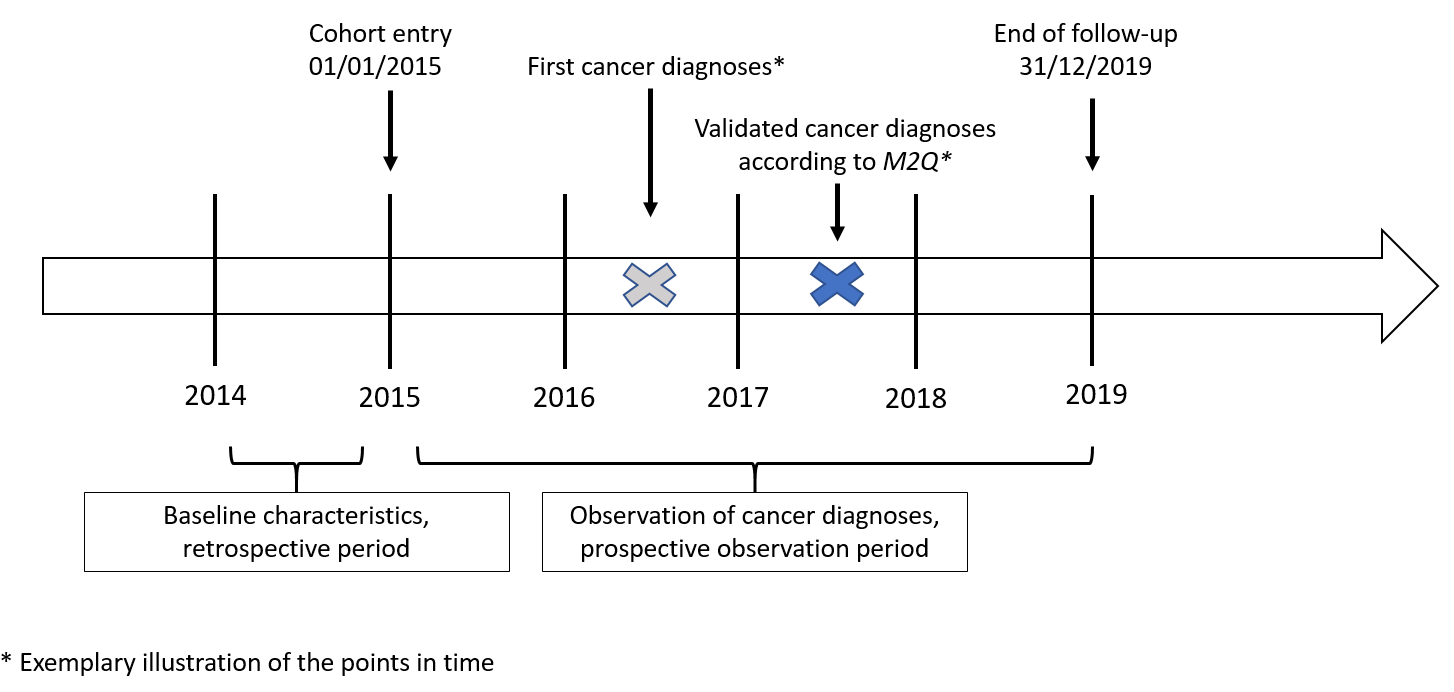
Figure S1: Timeline of cohort entry and observation period, Germany 2014 to 2019


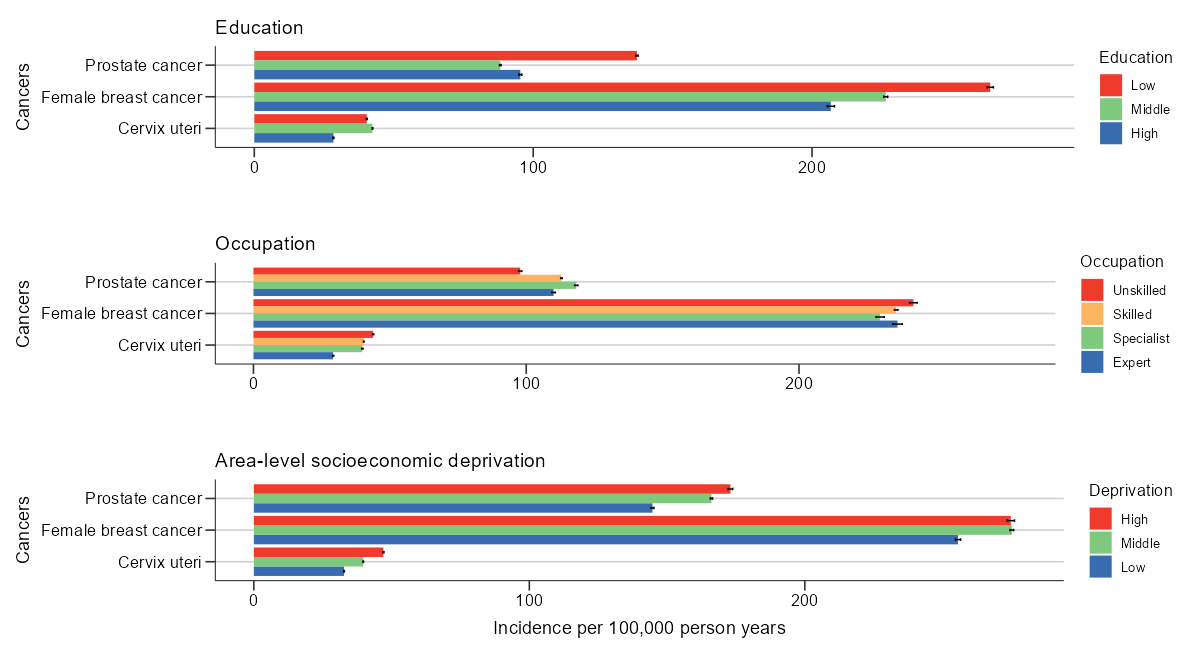


Figure S2: Sex-specific cancer incidence by education, occupation and deprivation in the German statutorily health-insured working age-population, Germany 2015-2019

Table S1: Distribution of the 2013 European Standard Population (ESP)

| Age group | ESP | Age-specific weight |
| --- | --- | --- |
| 25-29 | 6,000 | 0.11 |
| 30-34 | 6,500 | 0.12 |
| 35-39 | 7,000 | 0.12 |
| 40-44 | 7,000 | 0.12 |
| 45-49 | 7,000 | 0.12 |
| 50-54 | 7,000 | 0.12 |
| 55-59 | 6,500 | 0.12 |
| 60-64 | 6,000 | 0.11 |
| 65-67 | 3,300 | 0.06 |

Table S2: Hazard ratios (95% CI) of sex-specific cancer by socioeconomic factors among statutorily health-insured working age-population, Germany 2015-2019

|  | Female breast cancer | | Cervix uteri | | Prostate cancer | |
| --- | --- | --- | --- | --- | --- | --- |
|  | Step 1  HR and 95% CI | Step 2  HR and 95% CI | Step 1  HR and 95% CI | Step 2  HR and 95% CI | Step 1  HR and 95% CI | Step 2  HR and 95% CI |
| Considered cases | n=14, 208 | n=14,208 | n=2,096 | n=2,096 | n=8,568 | n=8,568 |
| Education | Model 1a | Model 2 | Model 1a | Model 2 | Model 1a | Model 2 |
| Low | 0.9* (0.8-0.9) | 0.9* (0.8-1.0) | 1.6* (1.3-1.9) | 1.4* (1.2-1.8) | 0.8* (0.7-0.9) | 0.8* (0.7-0.9) |
| Middle | 0.9*(0.9-1.0) | 1.0 (0.9-1.0) | 1.6* (1.3-1.9) | 1.4* (1.2-1.8) | 0.8* (0.8-0.9) | 0.9* (0.8-1.0) |
| High (ref) |  |  |  |  |  |  |
| Missings | 0.9* (0.8-0.9) | 0.9 (0.8-1.0) | 1.6* (1.3-1.9) | 1.4* (1.1-1.8) | 0.8* (0.7-0.9) | 0.8* (0.7-0.9) |
| Occupation | Model 1b |  | Model 1b |  | Model 1b |  |
| Unskilled | 0.8* (0.8-0.9) | 0.9* (0.8-1.0) | 1.6* (1.3-2.0) | 1.3 (1.0-1.7) | 0.8* (0.7-0.9) | 0.9 (0.8-1.0) |
| Skilled | 0.9* (0.8-1.0) | 0.9 (0.9-1.0) | 1.4* (1.2-1.8) | 1.2 (0.9-1.5) | 0.9* (0.8-1.0) | 1.0 (0.9-1.1) |
| Specialist | 0.9 (0.8-1.0) | 0.9 (0.9-1.0) | 1.4* (1.1-1.8) | 1.2 (0.9-1.6) | 1.0 (0.9-1.1) | 1.1 (1.0-1.2) |
| Expert (ref) |  |  |  |  |  |  |
| Missings | 0.9* (0.8-0.9) | 0.9 (0.8-1.0) | 1.5* (1.2-1.9) | 1.3 (0.9-1.7) | 0.9* (0.8-1.0) | 1.0 (0.9-1.2) |
| Area-level socioeconomic deprivation | Model 1c |  | Model 1c |  | Model 1c |  |
| High | 1.0 (0.9-1.1) | 1.0 (0.9-1.1) | 1.4* (1.2-1.6) | 1.4* (1.1-1.6) | 1.0 (0.9-1.1) | 1.0 (0.9-1.1) |
| Middle | 1.0 (1.0-1.1) | 1.0 (1.0-1.1) | 1.2* (1.1-1.4) | 1.2* (1.0-1.4) | 1.0 (1.0-1.1) | 1.1 (1.0-1.1) |
| Low (ref) |  |  |  |  |  |  |
| Missings | 1.1 (0.8-1.6) | 1.1 (0.8-1.6) | 1.8 (1.0-3.4) | 1.8 (1.0-3.4) | 0.9 (0.6-1.3) | 0.9 (0.6-1.3) |

* Statistically significant (p-value ≤ 0,05)

Step 1: separate models with adjustment for age
Step 2: full model with adjustment for age and mutual adjustment for education, occupation and area-level socioeconomic deprivation

Table S3: Variance Inflation Factor of all regression models

|  | **Women** | | | | **Men** | | | |
| --- | --- | --- | --- | --- | --- | --- | --- | --- |
| **Stomach cancer** | **M1a** | **M1b** | **M1c** | **M2a** | **M1d** | **M1e** | **M1f** | **M2b** |
| Age | 1.02 | 1.02 | 1.00 | 1.02 | 1.02 | 1.03 | 1.00 | 1.03 |
| Education | 1.05 |  |  | 1.30 | 1.05 |  |  | 1.34 |
| Occupation |  | 1.04 |  | 1.23 |  | 1.05 |  | 1.25 |
| Area-level deprivation |  |  | 1.00 | 1.00 |  |  | 1.00 | 1.00 |
| **Colorectal cancer** | **M1a** | **M1b** | **M1c** | **M2a** | **M1d** | **M1e** | **M1f** | **M2b** |
| Age | 1.02 | 1.02 | 1.00 | 1.02 | 1.02 | 1.03 | 1.00 | 1.03 |
| Education | 1.05 |  |  | 1.36 | 1.06 |  |  | 1.37 |
| Occupation |  | 1.04 |  | 1.26 |  | 1.05 |  | 1.28 |
| Area-level deprivation |  |  | 1.00 | 1.00 |  |  | 1.00 | 1.00 |
| **Lung cancer** | **M1a** | **M1b** | **M1c** | **M2a** | **M1d** | **M1e** | **M1f** | **M2b** |
| Age | 1.01 | 1.02 | 1.00 | 1.02 | 1.02 | 1.02 | 1.00 | 1.02 |
| Education | 1.04 |  |  | 1.34 | 1.05 |  |  | 1.33 |
| Occupation |  | 1.04 |  | 1.25 |  | 1.05 |  | 1.25 |
| Area-level deprivation |  |  | 1.00 | 1.00 |  |  | 1.00 | 1.00 |
| **Malignant melanoma of the skin** | **M1a** | **M1b** | **M1c** | **M2a** | **M1d** | **M1e** | **M1f** | **M2b** |
| Age | 1.02 | 1.02 | 1.00 | 1.02 | 1.02 | 1.03 | 1.00 | 1.03 |
| Education | 1.05 |  |  | 1.36 | 1.06 |  |  | 1.40 |
| Occupation |  | 1.04 |  | 1.27 |  | 1.05 |  | 1.29 |
| Area-level deprivation |  |  | 1.00 | 1.00 |  |  | 1.00 | 1.00 |
| **Female breast cancer** | **M1a** | **M1b** | **M1c** | **M2a** | **M1d** | **M1e** | **M1f** | **M2b** |
| Age | 1.02 | 1.02 | 1.00 | 1.02 | - | - | - | - |
| Education | 1.05 |  |  | 1.36 | - | - | - | - |
| Occupation |  | 1.04 |  | 1.27 | - | - | - | - |
| Area-level deprivation |  |  | 1.00 | 1.00 | - | - | - | - |
| **Cervix Uteri** | **M1a** | **M1b** | **M1c** | **M2a** | **M1d** | **M1e** | **M1f** | **M2b** |
| Age | 1.01 | 1.02 | 1.00 | 1.02 | - | - | - | - |
| Education | 1.04 |  |  | 1.31 | - | - | - | - |
| Occupation |  | 1.03 |  | 1.23 | - | - | - | - |
| Area-level deprivation |  |  | 1.00 | 1.00 | - | - | - | - |
| **Prostate cancer** | **M1a** | **M1b** | **M1c** | **M2a** | **M1d** | **M1e** | **M1f** | **M2b** |
| Age | - | - | - | - | 1.03 | 1.03 | 1.00 | 1.03 |
| Education | - | - | - | - | 1.06 |  |  | 1.42 |
| Occupation | - | - | - | - |  | 1.06 |  | 1.31 |
| Area-level deprivation | - | - | - | - |  |  | 1.00 | 1.00 |

Note: scaled as Generalized Variance Inflation Factor = GVIF^(1 / (2 * Df))

Table S4: Hazard ratios (95% CI) of cancer by socioeconomic factors among the insured working age-population, Germany 2015-2019 (Complete Cases Analysis)

|  | Stomach cancer | | Colorectal cancer | | Lung cancer | | Malignant melanoma of the skin | |
| --- | --- | --- | --- | --- | --- | --- | --- | --- |
|  | Step 1  HR and 95% CI | Step 2  HR and 95% CI | Step 1  HR and 95% CI | Step 2  HR and 95% CI | Step 1  HR and 95% CI | Step 2  HR and 95% CI | Step 1  HR and 95% CI | Step 2  HR and 95% CI |
| **Women** |  |  |  |  |  |  |  |  |
| **Education** | **Model 1a (n=292)** | **Model 2a (n=286)** | **Model 1a (n=1,805)** | **Model 2a (n=1,779)** | **Model 1a (n=1,074)** | **Model 2a (n=1,062)** | **Model 1a (n=2,615)** | **Model 2a (n=2,559)** |
| Low | 1.3 (0.8-1.9) | 1.0 (0.6-1.6) | 1.1 (1.0-1.4) | 1.3* (1.1-1.6) | 2.4* (1.8-3.3) | 2.4* (1.7-3.3) | 0.9 (0.8-1.1) | 1.0 (0.9-1.2) |
| Medium | 1.0 (0.7-1.6) | 0.9 (0.5-1.3) | 1.0 (0.9-1.2) | 1.1 (1.0-1.4) | 1.7* (1.2-2.2) | 1.7* (1.2-2.3) | 1.0 (0.9-1.2) | 1.1 (1.0-1.3) |
| High (ref) |  |  |  |  |  |  |  |  |
| **Occupation** | **Model 1b (n=350)** |  | **Model 1b (n=2,043)** |  | **Model 1b (n=1,255)** |  | **Model 1b (n=2,836)** |  |
| Unskilled | 1.7* (1.0-2.9) | 1.3 (0.7-2.5) | 0.9 (0.7-1.0) | 0.8* (0.6-0.9) | 1.8* (1.4-2.4) | 1.2 (0.9-1.6) | 0.6* (0.5-0.7) | 0.6* (0.5-0.7) |
| Skilled | 1.5 (1.0-2.4) | 1.6 (0.9-2.7) | 0.8* (0.7-1.0) | 0.8* (0.6-0.9) | 1.3 (1.0-1.6) | 0.9 (0.7-1.2) | 0.9 (0.8-1.0) | 0.9 (0.8-1.1) |
| Specialist | 0.9 (0.5-1.7) | 1.0 (0.5-1.9) | 0.8 (0.7-1.0) | 0.8* (0.6-1.0) | 1.0 (0.8-1.4) | 0.9 (0.6-1.2) | 1.0 (0.8-1.2) | 1.0 (0.8-1.2) |
| Expert (ref) |  |  |  |  |  |  |  |  |
| **Area-level deprivation** | **Model 1c (n=705)** |  | **Model 1c (n=3,867)** |  | **Model 1c (n=3,008)** |  | **Model 1c (n=4,018)** |  |
| High | 1.6* (1.2-2.1) | 1.4 (0.9-2.2) | 1.1 (0.9-1.3) | 1.1 (0.8-1.4) | 1.3* (1.1-1.5) | 1.3* (1.0-1.6) | 0.6* (0.5-0.7) | 0.7* (0.5-0.8) |
| Medium | 1.1 (0.9-1.4) | 1.1 (0.8-1.5) | 1.1 (1.0-1.3) | 1.1 (0.9-1.4) | 1.1* (1.0-1.3) | 1.1 (0.9-1.3) | 0.8* (0.7-0.9) | 0.9* (0.8-1.0) |
| Low (ref) |  |  |  |  |  |  |  |  |
| **Men** |  |  |  |  |  |  |  |  |
| **Education** | **Model 1d (n=747)** | **Model 2b (n=742)** | **Model 1d (n=2,808)** | **Model 2b (n=2776)** | **Model 1d (n=2,020)** | **Model 2b (n=1,997)** | **Model 1d (n=2,274)** | **Model 2b (n=2,242)** |
| Low | 1.5* (1.2-2.0) | 1.2 (0.9-1.6) | 1.1 (1.0-1.3) | 1.1 (0.9-1.3) | 2.9* (2.3-3.6) | 2.0* (1.6-2.5) | 0.6* (0.5-0.7) | 0.7* (0.6-0.8) |
| Medium | 1.3 (0.9-1.7) | 1.0 (0.8-1.4) | 1.1 (0.9-1.2) | 1.1 (0.9-1.2) | 2.0* (1.6-2.5) | 1.5* (1.2-1.9) | 0.7* (0.6-0.8) | 0.8* (0.7-0.9) |
| High (ref) |  |  |  |  |  |  |  |  |
| **Occupation** | **Model 1e (n=834)** |  | **Model 1e**  **(n=3,069)** |  | **Model 1e (n=2,293)** |  | **Model 1e (n=2,428)** |  |
| Unskilled | 1.7* (1.2-2.2) | 1.6* (1.1-2.3) | 1.1 (0.9-1.2) | 1.0 (0.9-1.2) | 2.9* (2.4-3.5) | 2.0* (1.6-2.6) | 0.6* (0.5-0.7) | 0.7* (0.6-0.8) |
| Skilled | 1.4* (1.1-1.8) | 1.3 (1.0-1.8) | 1.1 (0.9-1.2) | 1.0 (0.9-1.2) | 2.3* (1.9-2.7) | 1.7* (1.4-2.1) | 0.7* (0.6-0.7) | 0.8* (0.7-0.9) |
| Specialist | 1.3 (0.9-1.7) | 1.2 (0.9-1.7) | 1.0 (0.9-1.1) | 1.0 (0.8-1.1) | 1.3* (1.1-1.6) | 1.1 (0.9-1.4) | 0.9 (0.8-1.0) | 1.0 (0.9-1.2) |
| Expert (ref) |  |  |  |  |  |  |  |  |
| **Area-level deprivation** | **Model 1f (n=1,325)** |  | **Model 1f (n=4,671)** |  | **Model 1f (n=4,211)** |  | **Model 1f (n=3,259)** |  |
| High | 1.2* (1.0-1.4) | 1.4* (1.1-1.8) | 1.0 (0.9-1.2) | 1.1 (0.9-1.3) | 1.5* (1.3-1.7) | 1.4* (1.2-1.6) | 0.6* (0.5-0.6) | 0.6* (0.5-0.8) |
| Medium | 1.4* (1.2-1.6) | 1.2* (1.0-1.5) | 1.1 (1.0-1.3) | 1.2* (1.1-1.4) | 1.3* (1.1-1.4) | 1.2* (1.0-1.3) | 0.7* (0.7-0.8) | 0.8* (0.7-0.9) |
| Low (ref) |  |  |  |  |  |  |  |  |

*statistically significant (p-value ≤ 0,05)

Step 1: sex-specific models 1a to 1f are six independently estimated models in which each of the three SEP indicators used was separately adjusted for age.
Step 2: sex-specific models 2a and 2b with adjustment for age and mutual adjustment for education, occupation and area-level socioeconomic deprivation

Table S5: Hazard ratios (95% CI) of sex-specific cancer by socioeconomic factors among statutorily health-insured working age-population, Germany 2015-2019 (Complete Cases Analysis)

|  | Female breast cancer | | Cervix uteri | | Prostate cancer | |
| --- | --- | --- | --- | --- | --- | --- |
|  | Step 1  HR and 95% CI | Step 2  HR and 95% CI | Step 1  HR and 95% CI | Step 2  HR and 95% CI | Step 1  HR and 95% CI | Step 2  HR and 95% CI |
| **Education** | **Model 1a (n=7,788)** | **Model 2a (n=7,662)** | **Model 1a (n=1,325)** | **Model 2a (n=1,281)** | **Model 1d (n=4,518)** | **Model 2b (n=4,482)** |
| Low | 0.9* (0.8-0.9) | 0.9* (0.8-1.0) | 1.6* (1.3-1.9) | 1.4* (1.1-1.8) | 0.8* (0.7-0.9) | 0.8* (0.7-0.9) |
| Middle | 0.9*(0.9-1.0) | 1.0 (0.9-1.0) | 1.6* (1.3-1.9) | 1.4* (1.2-1.8) | 0.8* (0.8-0.9) | 0.9* (0.8-1.0) |
| High (ref) |  |  |  |  |  |  |
| **Occupation** | **Model 1b (n=8,641)** |  | **Model 1b (n=1,468)** |  | **Model 1e (n=4,921)** |  |
| Unskilled | 0.8* (0.8-0.9) | 0.9* (0.8-1.0) | 1.6* (1.2-2.0) | 1.3 (1.0-1.7) | 0.8* (0.7-0.9) | 0.9 (0.8-1.0) |
| Skilled | 0.9* (0.8-1.0) | 0.9 (0.9-1.0) | 1.4* (1.2-1.8) | 1.2 (0.9-1.5) | 0.9* (0.8-1.0) | 1.0 (0.9-1.1) |
| Specialist | 0.9 (0.8-1.0) | 1.0 (0.9-1.1) | 1.4* (1.1-1.8) | 1.2 (0.9-1.6) | 1.0 (0.9-1.1) | 1.1 (1.0-1.3) |
| Expert (ref) |  |  |  |  |  |  |
| **Area-level socioeconomic deprivation** | **Model 1c (n=14,013)** |  | **Model 1c (n=2,035)** |  | **Model 1f (n=8,504)** |  |
| High | 1.0 (0.9-1.1) | 1.0 (0.9-1.1) | 1.4* (1.2-1.7) | 1.3* (1.1-1.6) | 1.0 (0.9-1.1) | 1.0 (0.9-1.2) |
| Middle | 1.0 (1.0-1.1) | 1.0 (1.0-1.1) | 1.2* (1.1-1.4) | 1.2* (1.0-1.4) | 1.0 (1.0-1.1) | 1.1 (1.0-1.2) |
| Low (ref) |  |  |  |  |  |  |

* Statistically significant (p-value ≤ 0,05)

Step 1: separate models with adjustment for age
Step 2: full model with adjustment for age and mutual adjustment for education, occupation and area-level socioeconomic deprivation

Table S6: Variance Inflation Factor of all regression models (Complete Cases Analysis)

|  | **Women** | | | | **Men** | | | |
| --- | --- | --- | --- | --- | --- | --- | --- | --- |
| **Stomach cancer** | **M1a** | **M1b** | **M1c** | **M2a** | **M1d** | **M1e** | **M1f** | **M2b** |
| Age | 1.00 | 1.02 | 1.00 | 1.00 | 1.00 | 1.03 | 1.00 | 1.00 |
| Education | 1.02 |  |  | 1.08 | 1.01 |  |  | 1.08 |
| Occupation |  | 1.04 |  | 1.03 |  | 1.05 |  | 1.04 |
| Area-level deprivation |  |  | 1.00 | 1.00 |  |  | 1.00 | 1.00 |
| **Colorectal cancer** | **M1a** | **M1b** | **M1c** | **M2a** | **M1d** | **M1e** | **M1f** | **M2b** |
| Age | 1.00 | 1.02 | 1.00 | 1.00 | 1.00 | 1.03 | 1.00 | 1.00 |
| Education | 1.01 |  |  | 1.09 | 1.01 |  |  | 1.09 |
| Occupation |  | 1.04 |  | 1.04 |  | 1.05 |  | 1.04 |
| Area-level deprivation |  |  | 1.00 | 1.00 |  |  | 1.00 | 1.00 |
| **Lung cancer** | **M1a** | **M1b** | **M1c** | **M2a** | **M1d** | **M1e** | **M1f** | **M2b** |
| Age | 1.00 | 1.02 | 1.00 | 1.00 | 1.00 | 1.02 | 1.00 | 1.00 |
| Education | 1.01 |  |  | 1.07 | 1.00 |  |  | 1.06 |
| Occupation |  | 1.04 |  | 1.03 |  | 1.05 |  | 1.03 |
| Area-level deprivation |  |  | 1.00 | 1.00 |  |  | 1.00 | 1.00 |
| **Malignant melanoma of the skin** | **M1a** | **M1b** | **M1c** | **M2a** | **M1d** | **M1e** | **M1f** | **M2b** |
| Age | 1.00 | 1.02 | 1.00 | 1.00 | 1.00 | 1.03 | 1.00 | 1.00 |
| Education | 1.01 |  |  | 1.09 | 1.01 |  |  | 1.11 |
| Occupation |  | 1.04 |  | 1.04 |  | 1.05 |  | 1.05 |
| Area-level deprivation |  |  | 1.00 | 1.00 |  |  | 1.00 | 1.00 |
| **Female breast cancer** | **M1a** | **M1b** | **M1c** | **M2a** | **M1d** | **M1e** | **M1f** | **M2b** |
| Age | 1.00 | 1.02 | 1.00 | 1.00 | - | - | - | - |
| Education | 1.01 |  |  | 1.09 | - | - | - | - |
| Occupation |  | 1.04 |  | 1.04 | - | - | - | - |
| Area-level deprivation |  |  | 1.00 | 1.00 | - | - | - | - |
| **Cervix Uteri** | **M1a** | **M1b** | **M1c** | **M2a** | **M1d** | **M1e** | **M1f** | **M2b** |
| Age | 1.00 | 1.02 | 1.00 | 1.00 | - | - | - | - |
| Education | 1.01 |  |  | 1.09 | - | - | - | - |
| Occupation |  | 1.03 |  | 1.04 | - | - | - | - |
| Area-level deprivation |  |  | 1.00 | 1.00 | - | - | - | - |
| **Prostate cancer** | **M1a** | **M1b** | **M1c** | **M2a** | **M1d** | **M1e** | **M1f** | **M2b** |
| Age | - | - | - | - | 1.00 | 1.03 | 1.00 | 1.00 |
| Education | - | - | - | - | 1.00 |  |  | 1.09 |
| Occupation | - | - | - | - |  | 1.06 |  | 1.04 |
| Area-level deprivation | - | - | - | - |  |  | 1.00 | 1.00 |

Note: scaled as Generalized Variance Inflation Factor = GVIF^(1 / (2 * Df))
